# P300 and Decision Making under Risk and Ambiguity

**DOI:** 10.1155/2015/108417

**Published:** 2015-10-11

**Authors:** Lei Wang, Jiehui Zheng, Shenwei Huang, Haoye Sun

**Affiliations:** ^1^Department of Management Science and Engineering, School of Management, Zhejiang University, Hangzhou 310058, China; ^2^Neuromanagement Lab, Zhejiang University, Hangzhou 310027, China

## Abstract

Our study aims to contrast the neural temporal features of early stage of decision making in the context of risk and ambiguity. In monetary gambles under ambiguous or risky conditions, 12 participants were asked to make a decision to bet or not, with the event-related potentials (ERPs) recorded meantime. The proportion of choosing to bet in ambiguous condition was significantly lower than that in risky condition. An ERP component identified as P300 was found. The P300 amplitude elicited in risky condition was significantly larger than that in ambiguous condition. The lower bet rate in ambiguous condition and the smaller P300 amplitude elicited by ambiguous stimuli revealed that people showed much more aversion in the ambiguous condition than in the risky condition. The ERP results may suggest that decision making under ambiguity occupies higher working memory and recalls more past experience while decision making under risk mainly mobilizes attentional resources to calculate current information. These findings extended the current understanding of underlying mechanism for early assessment stage of decision making and explored the difference between the decision making under risk and ambiguity.

## 1. Introduction

Risk and ambiguity are two conditions in which the likelihood of outcomes is uncertain [1]. But differences are here to stay; in the condition of risk, the probability distribution of possible outcomes is well defined, which can be used to calculate the expectancies of outcomes and compare between choices. The probability of outcomes determines the riskiness of risk condition that high probability brings lower risk and vice versa. However, under the ambiguous condition, participants are unknown of the probabilities of outcome [2–4]. The participants tend to subjectively add probability of the outcome in decision making, and it is difficult to be described by the theoretical models accurately. The distinction between the two uncertain conditions was first illustrated by the Ellsberg Paradox, which indicates the so-called phenomenon of ambiguity aversion [5] that means the peoples' preference to bet in risky conditions rather than ambiguous conditions.

Researchers have investigated the underlying mechanism of ambiguity aversion for a long time and put forward some explanations, such as the competence hypothesis [5] and the comparative ignorance [6]. Rode and colleagues put these hypotheses into two categories: cognitive approach and motivational approach [7]. Cognitive mechanism regarded ambiguity as a second-order probability distribution of option [8, 9]. Participants can obtain probability information from former experience. Motivational approach focused on effective factors that come from the lack of information. Frisch and Baron put forward that the risky prospect is more justifiable than the ambiguous one due to missing potentially available probabilistic information of the latter [10]. Fox and Weber found people feeling less confident for issues that they did not fully understand [11]. And a bad outcome in ambiguous conditions may be ascribed to the incompetence or thoughtless choice [12], while in risky conditions the poor judgment cannot be blamed for when the outcome is undesirable. Since all required information is provided, participants are more likely to attribute the bad outcome to bad luck [5].

In the latest ten years, numerous empirical studies about uncertainty decision making using functional magnetic resonance imaging (fMRI) tools have attempted to contrast these two types of uncertainty at neural level, and identified the neural mechanism related to decision making under ambiguity and risk [3, 13–15]. Hsu et al. suggested a general neural circuit responding to degrees of uncertainty [14]. In their first treatment of Card-Deck that compared the pure risk (where probabilities were known with certainty) against pure ambiguity as baseline, the participants were asked to choose between betting on one of the two options and taking a fixed monetary reward in each trail. Finally, they compared the difference between ambiguity and certain conditions, and difference between risk and certain conditions, respectively. However, in our experiment, we asked the participants to choose to bet or not in ambiguous and risky conditions, in both of which they would get no fixed payouts if they gave up betting. This design made us compare the difference of decision making between risky and ambiguous conditions, which was not further studied in Hsu et al.'s work [14].

Compared with previous fMRI studies [3, 4], event-related brain potentials (ERPs) offer better temporal resolutions for researchers to study how a cognitive process is taking place in real-time. Decision making is a continuous process, which can be divided into several stages temporally, including assessment and formation of preferences among possible options, selection, and feedback or evaluation of an outcome. Until now, a lot of work has been conducted for the feedback stage of decision making while little attention is allocated to earlier stages, such as assessment of the options. Previous ERP studies about decision making under uncertainty mainly focused on the feedback stage and explored components such as FRN and P300 [16–19]. Zhang's studies indicated that the P300 component was sensitive to risky decision making [20]. Zhou et al. used a risky gambling game and found out that the P300 and FRN were quite different between the conditions of win and loss [21]. Xu and her colleagues applied event-related brain potential (ERP) to explore how an uncertain (risk and ambiguity) cue was processed. They designed a gambling task called “wheel of fortune” and found out that a larger P300 was elicited by the unexpected cue under uncertain condition [22]. Gu and his colleagues found that P3 was larger in the positive outcome condition than the other three conditions (negative, neutral, and ambiguous) by using a monetary gambling task [23]. These studies revealed that both the risky and ambiguous conditions would evoke the P300. Previous studies, including the above two studies, mainly focused on the feedback stage and viewed P300 as a typical indicator to reflect rewarding processing in decision-making [19, 24, 25]. However, until now few ERP studies have focused on the early stage of decision making before the feedback stage and explored the corresponding neural mechanism.

P300 is one of the most commonly studied components of ERPs for decision making [26], and it usually emerges in the late period after stimuli onset (300–600 ms). In task relevant paradigms, the amplitude of P300 is generally considered as a representation of memory load [27–29]. Task load can be divided into two dimensions: driving task load and working memory load [30]. P300 is related to memory processes in the evaluation of stimuli for the subsequent response [31, 32]. Task-related information is updated through learning and forgetting in working memory and P300 is elicited at the same time [33]. The decrease of P300 amplitude was observed in several tasks employing high memory load [34–36]. Since ambiguous tasks provide less definite information than risky ones, individuals need to not only mobilize some attentional resources to analyze current stimuli, but also recall a large amount of past practices and memory to get probability information to form a clear expectance of outcome and reduce cognitive strain in dealing with ambiguous tasks. So it is more effortful and difficult for participants to make decisions under the ambiguous condition, which may induce a higher working memory load [37, 38].

Compared with the previous studies, the present research focuses on the temporal electrophysiological changes and difference at the early stimuli assessment stage of decision making between the ambiguous and risky conditions, which we believe can help better understand the process of decision making. For exploring the cognitive process of these two decision types, we designed a monetary gambling game in which the probabilities of outcomes were known (risk) or unknown (ambiguity) while the outcomes were varied across trials but balanced in pairs. Considering that the P300 is a typical neural indicator of decision making and can be interpreted as a reflection of memory load and is inversely related to difficulty of decision making [37, 38], we speculated that it would elicit a smaller P300 amplitude under ambiguity than that under risk decision making.

## 2. Materials and Methods

### 2.1. Participants

Participants were recruited from the student population of the Zhejiang University. A total number of 12 right-handed participants took part in the experiment (5 females; average age: 22.58 years, standard deviation (SD) = 1.55 years, range 20–25 years). All participants had no history of neurological or psychiatric disorders.

### 2.2. Materials

Prior to the experiment, participants were informed that the purpose of the experiment was to investigate brain waves during gambling. An informed consent, approved by the Internal Review Board of Neuromanagement Lab, Zhejiang University, was obtained from each participant before formal experiment.

**Figure 1:**
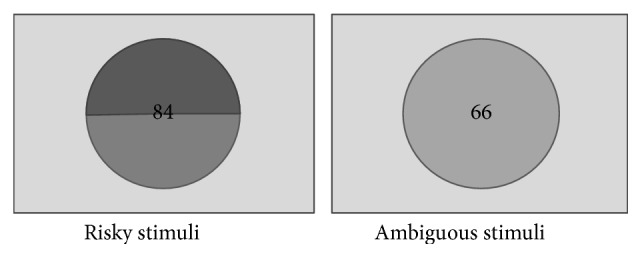
Stimulus: risk stimuli and ambiguous stimuli.

The game consists of two types of primes: risky stimuli and ambiguous stimuli. Risky stimuli presented a monetary value with 50% probability for gain and loss each. Ambiguous stimuli showed only a monetary value but concealed its corresponding probability on gain or loss. Risky stimuli and ambiguous stimuli appeared in the experiment with equal frequency. The monetary value was a random integer ranging from 11 to 190 for each trail in both conditions. Each stimulus was made into a picture and digitized at 200 × 150 pixels (Figure 1).

### 2.3. Procedure

**Figure 2:**
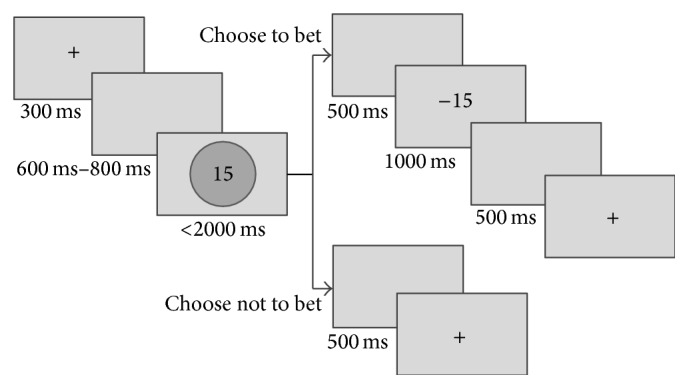
Task procedure.

The EEG participant was seated in a chair 1 meter in front of a Dell 22 in. CRT display (screen resolution: 1024 × 768; refresh rate: 120 Hz; color quality: highest 32 bit). Stimuli were presented sequentially in the center of a computer screen with a visual angle of 2.58° × 2.4°. In order to draw participants' attention, the screen presents a “+” for a fixed duration of 300 ms at the beginning of each trial. Then, a stimulus of risk or ambiguity was shown after a mean delay of 700 ms. After the presentation of the risky or ambiguous stimuli, if the participants chose to bet, a 500 ms blank was presented followed by the outcome with 50% probability of lose or win for 1000 ms; otherwise it went to the next trial after a 500 ms blank (see Figure 2).

All subjects were asked to read the experiment instructions before the experiment. A practice block was administered before the formal test. The formal test had 6 blocks with 60 trials each. Each condition (risk versus ambiguity) had 180 trials distributed randomly in 6 blocks. Each gambling value was presented twice: one in risky condition and the other in ambiguous condition. In half of the trials in each condition, the feedback was positive (the participants would gain if they decided to bet); in the other half of trials, the feedback was negative. The total gains and total losses were counterbalanced in each condition and across the two conditions. However, the participants were blind to this design; they were only told to choose to bet or not and that they would be rewarded according to their performance. The presentation of stimuli and recording of the participant's responses were controlled by STIM 2 software (Stim2, Neurosoft Labs, Inc., Sterling, USA).

In summary, all the stimuli probabilities and the monetary magnitude presented in risk and ambiguity conditions were the same in our experiment design. The only difference between risk and ambiguity conditions was whether the participants knew the probability of outcome. Before the experiment, the participants were told that their final payment was decided by their performance in the experiment. That is, 35-Yuan basic payments for their participation and an additional gain or loss based on the mean of total investment outcomes, by which means we motivated the participants to make decisions carefully and effectively in all trails in order to achieve the maximum benefit.

### 2.4. Electroencephalography (EEG) Recording

The EEG and the EOG (electrooculograms) were recorded and preprocessed by Neuroscan Synamp2 Amplifier (Scan4.5, Neurosoft Labs, Inc., Sterling, USA), with the reference to the left mastoid. In order to keep the impedances of electrodes below 5 kOhm all electrode sites were cleaned with electrode jelly and gently abraded prior to electrode fixation. EEG and EOG were amplified with a 64 channel AC amplifier (input impedance: 10 MOhm). Vertical Electrooculogram (EOG) was recorded supra and infra-orbitally at the left eye. Horizontal electrooculograms were recorded from electrodes placed 1.5 cm lateral to the left and right external canthi. Band-pass was set to 0.05–100 Hz; the signals were digitized online at 500 Hz and stored for later analyses.

Electroencephalogram recordings were segmented for the epoch from 200 ms before appearance of the stimulus picture for decision type to 800 ms after the stimulus onset, with the first 200 ms pretargets as a baseline. Trails were contaminated by amplifier clipping, wherein bursts of electromyography activity and peak-to-peak deflection exceeding ±80 *μ*V were excluded. Finally, EEG waveforms were averaged separately for each participant, each experimental condition, and each electrode. In addition, SPSS statistical software (SPSS Inc., SPSS Inc., Chicago, Illinois, USA) was used for data statistical analyses.

## 3. Results

### 3.1. Behavioral Data

**Figure 3:**
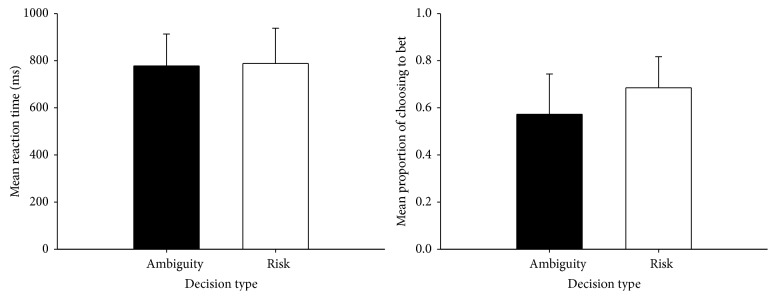
Behavioral data: reaction time and proportion of choosing to bet for decision under ambiguity and risk.

Behavior data are shown in Figure 3. A paired-sample *t*-test showed that there was no significant difference between the two decision types of ambiguity and risk about reaction time (RTs), *t* = −0.800, *P* = 0.439 > 0.05. In contrast to the RTs, the mean proportion of choosing to bet in ambiguous condition (57.24%, SD = 17.09%) was less than that in risky condition (68.49%, SD = 13.17%). A paired-sample *t*-test showed that the difference was significant (*t* = −2.250, *P* = 0.044 < 0.05). The results indicated that participants would rather bet in risky condition than bet in ambiguous condition, but it took them the same time to decide whether or not to bet in different conditions.

### 3.2. ERP Analyses

**Figure 4:**
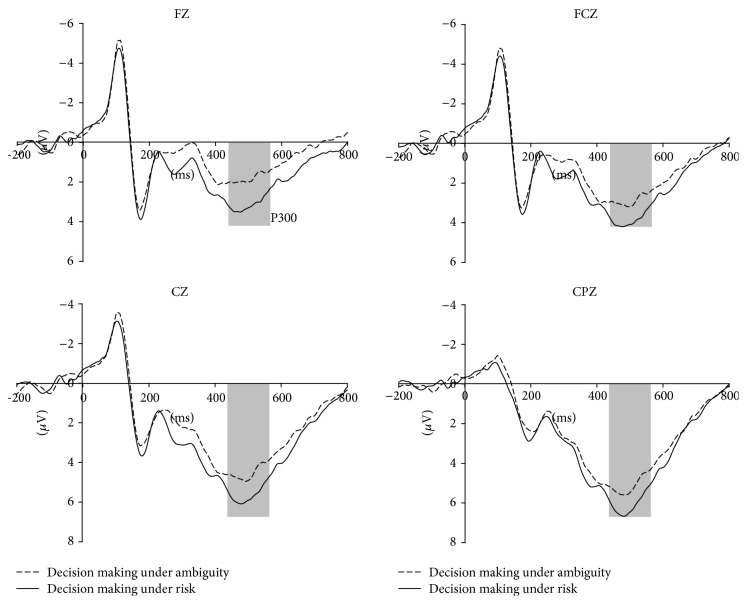
Grand averaged ERP waveforms for two stimulus conditions at electrode sites FZ, FCZ, CZ, and CPZ.

The component P300 was analyzed as well (Figure 4). The P300 amplitude, peaking at approximately 500 ms after stimulus onset, is mainly distributed in the center scalp areas [32, 39, 40]. Similar to the previous studies, this study selected 9 electrode sites (FC3, FCZ, FC4, C3, CZ, C4, CP3, CPZ, and CP4) for statistical analysis. Two-way repeated measure ANOVA testing across two levels of decision types and nine levels of electrodes were computed on P300 amplitude.

We measured the P300 average amplitudes in the shaded 450–550 ms time window for both risky and ambiguous conditions (in Figure 4). A 2 (decision type: risk and ambiguity) × 9 (electrode: FC3, FCZ, FC4, C3, CZ, C4, CP3, CPZ, and CP4) with subjects repeated measure ANOVA showed that there was a main effect for decision type [*F*(1,11) = 17.147, *P* = 0.002 < 0.05]. The grand average amplitude of 9 electrodes of risky condition (*M* = 5.595 *μ*V) was significantly larger than that of ambiguous condition (*M* = 4.513 *μ*V).

## 4. Discussion

Our behavioral data indicated ambiguity aversion with the results that the bet rate in risky condition was significantly higher than that in ambiguous condition. Besides, the ERP results showed that P300 amplitude elicited in ambiguous condition was significantly smaller than that evoked in risky condition, which revealed that higher working memory load was needed in the stage of assessment when making decision in ambiguous condition.

A large number of previous studies have demonstrated that people held different attitudes towards risk and ambiguity. According to Smith and colleagues [4], individual behavior may be affected by attitudes about payoffs (gains and losses) and beliefs about outcomes (risk and ambiguity). No matter the outcome is gain or loss, people are always ambiguity adverse. That is to say, people tend to be more adverse to ambiguity than to risk [5, 41, 42]. Thus our behavioral result showing that the proportion of subjects choosing to bet in ambiguous condition was significantly less than that in risky condition was consistent with the aforementioned studies [8, 9, 43].

P300 were elicited in both risky and ambiguous conditions, which was consistent with prior studies [22, 23]. Many studies related with decision making found that P300 was sensitive to many factors, such as the magnitude of reward [21, 44], the valence of reward [21], and interpersonal relationship in reward processing [18]. However, almost all of those studies focused on the feedback stage in which the features of reward matter. Compared with them, our experiment studied the P300 evoked in the stage of processing stimuli, which was earlier than feedback stage and did not involve the reward processing. Thus the factors influencing the modulation of P300 in our study were different from that in previous studies. Besides, we also controlled some other factors; that is, both the stimuli probabilities and the monetary magnitude presented in risky and ambiguous conditions were the same in our experiment design. The main difference between these two conditions was whether the probabilities were blind to the participants or not.

In gamble games in both risky and ambiguous conditions, people need to calculate the expectances of outcomes based on the probabilities of outcome and specific monetary value before they make a decision. In our experiment design, both of the two required pieces of information were provided in risky condition but only monetary value was given to participant in ambiguous condition. The lack of probabilities may induce more effort to recall a large amount of past practices and memory to get probability information to form a clear expectance of outcome and reduce cognitive strain in dealing with ambiguous tasks. As we know, learning from feedback plays a role in guiding decision making. Personal experience of similar situations has an effect on current decision. Positive or negative emotion induced from previous experience facilitates present information process [45]. In addition, working memory holds and manages information which exerts influence on subsequent behaviors in the short term [46]. Besides, such process of working memories appears universally in reality especially when dealing with decision making under incomplete information. According to previous studies, ambiguity can be regarded as a second-order probability distribution of option [8, 9] and people can obtain probability information from former experience. Thus, participants in ambiguous condition would learn the experience from former trails and infer the current probability, which induced a higher working memory load [38, 39]. But this process may not be expected in risky condition since the probability was definite and the outcomes were more likely to be attributed to the luck at present. Thus, we thought this difference resulted in the decrease of P300 amplitude in ambiguous condition.

Prior studies indicated that, in stimulus processing, the P300 could be considered as a representation of working memory [27–29] and it was widely demonstrated that the P300 amplitude is inversely proportional to working memory [34–36]. Thus, the lower P300 amplitude elicited in ambiguous condition revealed that participants employed higher memory load at assessment stage when they made decisions. Besides, as we know, the ambiguous decision making is relative to emotional process, and people would experience more negative emotion (such as being more worried and anxious) and be less confident, since they cannot exactly know the outcome [11]. As discussed above, people would need to trace back for evidences which required greater number of certainty cues in order to neutralize this negative feeling and make themselves confident under ambiguous conditions [47, 48]. They would not only pay attention to current stimuli (i.e., monetary value) as they did in risky condition, but also recall the past experience about the gamble to calculate a more clear expectance of outcome [49]. All in all, due to the integrality of information, it is much easier for decision maker to calculate expected value in risky condition than in ambiguous condition. Participants focus mainly on calculating explicit information for logical strategies in risky condition while they are more likely to mobilize past experience to figure out the possible probability [50] to reduce cognitive stress with insufficient information in ambiguous condition. This additional effort and difficulty resulted in a high working memory load with a lower P300 elicited. Therefore, our results of lower P300 amplitude in ambiguous condition than in risky one indicated the different cognitive mechanism between ambiguity and risk.

## 5. Conclusion

In this study, we used ERPs to clarify and extend the current understanding of decision making under risk and ambiguity. Particularly, our findings suggested that the P300 amplitude can be applied to reflect information processing at the early stage (the stage of assessment) of decision making. In our research, the P300 amplitude elicited by gambling in ambiguous condition was significantly smaller than that evoked in risky condition, showing that participants met with higher working memory under ambiguity to mobilize past experience to calculate the expected value and reduce cognitive strain than under risk. Furthermore, our behavioral result validated ambiguity aversion phenomenon with the finding of a lower bet rate in ambiguous condition.
